# Losing half the conductive area hardly impacts the water status of mature trees

**DOI:** 10.1038/s41598-018-33465-0

**Published:** 2018-10-09

**Authors:** Lars Dietrich, Günter Hoch, Ansgar Kahmen, Christian Körner

**Affiliations:** Department of Environmental Sciences - Botany, University of Basel, Schönbeinstrasse 6, CH-4056 Basel, Switzerland

## Abstract

The water status of transpiring tree crowns depends on a hydraulic continuum from the soil matrix around roots to the sub-stomatal cavity of leaves, with a multitude of hydraulic resistances along this path. Although the stem xylem path may not be the most critical of these resistances, it had been suggested that a >50% interruption of that path by drought-stress-induced embolization (air filling) of conduits is critical for tree survival. Here we show that cutting the sapwood of mature, 35 m tall trees in half hardly affects crown water status and transpiration. Counter expectation, this first adult tree sapwood interception experiment revealed that shoot water potential in the canopy (assessed by using a 45 m canopy crane) either remained unaffected (spruce) or became less negative (beech), associated with small reductions in leaf diffusive conductance for water vapour. We conclude that the stem xylem of these trees has a large overcapacity and the tree hydraulics debate requires a critical re-visitation.

## Introduction

Water transport in trees is ensured by the passive movement of water through capillary pipes in the xylem called conduits. Depending on species, these conduits vary in width, length, and connection with each other. Coniferous tree species possess tracheids only, which are elongated, individual cells that are interconnected by tiny pit pores in the cell wall. Angiosperm tree species, by contrast, feature both tracheids and tracheae, also called ‘vessels’. Vessels are larger diameter conduits composed of a whole series of cells that lost intermittent cell walls, with individual vessels connected by both, pits and so-called perforation plates, that allow water to easily pass from one vessel to the next^1^. Depending on their anatomy, tracheids and vessels reveal clearly different hydraulic properties^2^. Vessels allow for a higher conductivity at the price of a higher risk to embolize under capillary tension (vaporising of liquid water creating embolisms) which interrupts water flow under drought stress, often called hydraulic failure^3^.

The driving force of water movement through a tree is atmospheric evaporative demand (vapour pressure deficit) creating vapour loss from leaves (transpiration). Since water always flows from a point of higher water potential (soil) to a point of lower water potential (atmosphere) it moves through capillary conduits up the trunk across different resistances. The inter-fibril cohesion of water in transpiring cell walls in the leaf mesophyll is so strong that the cell wall cannot be dehydrated hydraulically, thus ensuring the capillary water continuum from root tips to the mesophyll surface of transpiring leaves. There are two, functionally very different reasons why water potential in the canopy can drop significantly below the hydrostatic potential (−0.1 MPa per 10 m height): (1) A high flux across a given series of resistances under good water supply causes negative water potentials (in the ‘physiological range’, commonly down to −1 to −2 MPa). The higher the hydraulic resistances are and the higher the flux, the steeper the drop of water potential in the canopy as a consequence of crown transpiration. (2) Under drought, the capillary continuum from the soil to the root surface becomes interrupted and water potentials in the canopy can become very negative (more negative than −2 MPa, down to −6 MPa), despite minute flux. Under such tension the conduits can fill with water vapour and air, causing hydraulic failure, which in turn is often assumed to cause tree mortality, a causality still lacking unambiguous evidential data in mature trees.

The hydraulic failure theory is based on the assumption that a loss of conductivity impairs the water supply to upstream plant organs, leading to tissue dehydration and eventually plant death. Water potentials measured in dying trees or saplings were found to be associated with a 50% loss of xylem hydraulic conductivity (50% loss of conductivity, PLC_50_)^4^. Thus, these water potentials at PLC_50_ (P_50_; or the very similar P_88_ in case of angiosperms) are thought to represent threshold values beyond which a tree will eventually die^5^, and were found to average around −3.6 MPa in woody angiosperms and around −5.6 MPa in gymnosperms^6^. The important point is that these water potentials and conduit losses occur in a situation in which sap flow is close to zero, that is, in a situation in which hardly any flux is required to balance cuticular water losses which commonly represent <1% of full transpiration in trees from environments with periodic drought^7^. From this point of view, it is rather unlikely that a 50% loss in stem conductivity can cause a critical branch water shortage when in fact, only around 1% of the conductive capacity would be required.

PLC threshold values are based on data either obtained from twigs of severely stressed trees in the field, the hydraulic conductivity of which was assessed in the lab^8,9^, or on experiments with pieces of twigs that were artificially embolized in a centrifuge that simulates increasing water tensions in conduits^10^. Yet, the direct causal relationship between PLC_50_ (or PLC_88_) and tree death has still not been established. A 50% loss of conductivity is commonly assumed to correspond to a 50% loss in conductive area (a proxy for xylem conduit water content), an assumption supported by micro CT imaging^11^. Yet, when flux approaches zero under severe drought (stomatal closure, leaf shedding), the demand in conduit capacity also approaches zero. In contrast, a situation in which trees run at close to maximum sap flow would require high conductivity, with a loss of half of all conduits potentially impacting crown water status severely. Admittedly, there may be qualitative differences between a chain saw cut and a diffuse blockade of 50% of conduit area during embolism events (as will be discussed later). Yet, the test had never been attempted in mature trees in the field and results of such a test, as presented here, are likely to advance the theory in this field.

**Figure 1:**
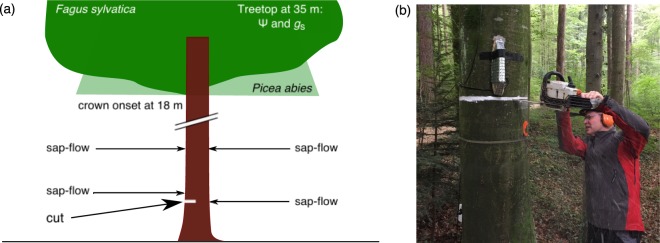
(**a**) Position of sap flow sensors that were installed before the treatment. (**b**) Interruption of half of all conductive sapwood on 14 August 2017 (DOY 226) with a chain saw. ©The authors.

To test the hypothesis that a 50% loss of conducting stem area leads to significant hydraulic constraints in transpiring trees, we selected eight similarly-sized tall trees, four from each of the two species *Fagus sylvatica* (European beech) and *Picea abies* (Norway spruce), which had a conducting sapwood width of 20–30 mm (ca. 10 annual rings; Supporting Fig. 1). In two individuals per species, we cut stems 65 mm deep, over half of their circumference 1.8 m above ground (at ca. 9 a.m. on a bright day in mid-August, DOY 226), with the other two individuals serving as controls (Fig. 1). For static reasons, we could not cut the entire half of the ca. 35 m tall stems, but the applied cutting depth was sufficient to reach well beyond the sapwood, and thus completely interrupted the water transport within half the stem (Supporting Fig. 1). The trees’ water status was monitored before, during, and after the cutting by sap flow gauges in the trunk, and by pressure chamber and porometer readings in the canopy (shoot water potential Ψ, and leaf diffusive conductance g), working from a canopy crane gondola. Sap flow sensors were installed in July (DOY 198) 10 cm centrally above the cut, directly opposite the cut, and 3 m above ground, axially aligned with the two lower-position sensors (Fig. 1). Control trees were equipped with two sensors at 1.8 m above ground, placed opposite from each other. In the treatment trees, we added another set of sap flow sensors horizontally aligned with the cut, 5 cm sideways from the edge of the cut, after the cut had been set in mid-September (DOY 263–268).

## Results

**Figure 2:**
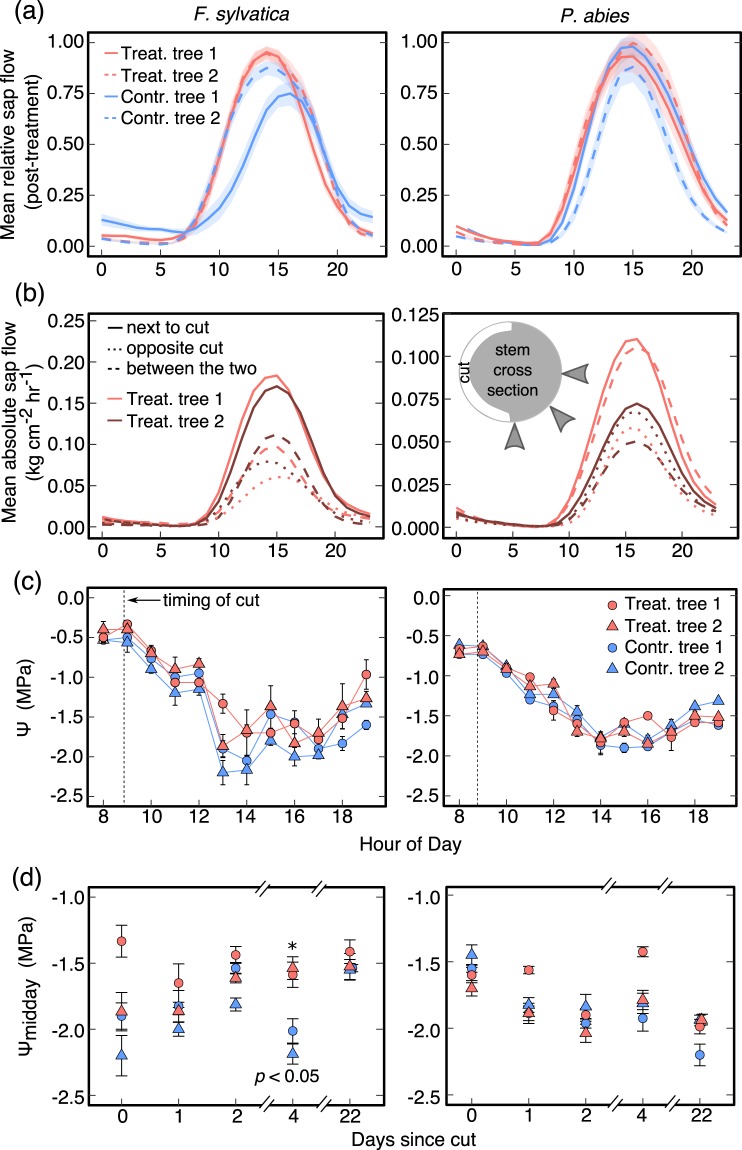
(**a**) Relative sap flow (half-hourly means) opposite the cut of treated and untreated trees during the first 18 days after cutting (n = 18 days ± SE). Sap flow was standardized by the mean of the maxima during the two-week pre-treatment period. (**b**) Mean absolute hourly sap flow of the subsequently installed sensors aligned sideways to the cut on 20 to 25 September 2017 (cut trees only). The inlet image shows the position of the sensors at the stem. (**c**) Shoot water potential on the first day of the experiment (14 August 2017; mean ± SE). Means for three sun-exposed current-year branches per tree per hour (n = 3). (**d**) Midday shoot water potential in the canopy after the cut was set (mean ± SE for three sun-exposed branches per hour and tree). The cut was set on day 0 at 9:10 am.

Before and after the treatment, the tree canopies were green and healthy without any foliage loss until autumn leaf shedding (in *Fagus)*. While sap flow above the cut was reduced to zero in both species following the treatment (Supporting Fig. 2), sap flow in *Fagus* opposite the cut increased slightly (around midday only) and remained unchanged in *Picea* (Fig. 2a). However, flow rates 5 cm next to the cut were 200% (*Fagus*) and 40% (*Picea*) higher than opposite the cut (Fig. 2b). Also, flow rates halfway between the sensors next to and opposite the cut were still up to 80% (*Fagus*) and 40% (*Picea*) higher than opposite the cut (Fig. 2b). The sensors 1.3 m vertically above the cut showed only about 10% of pre-treatment flow (Supporting Fig. 2).

Ψ in tree crowns on the day of the cut did not show any difference compared to control in *Picea* and became less negative in *Fagus* around 2 p.m. (Fig. 2c). Also during the following days after the cut, Ψ of both species did not drop to more negative values than in the control trees but rather persistently relaxed in *Fagus* even when control trees revealed very negative values (Fig. 2d).

**Figure 3:**
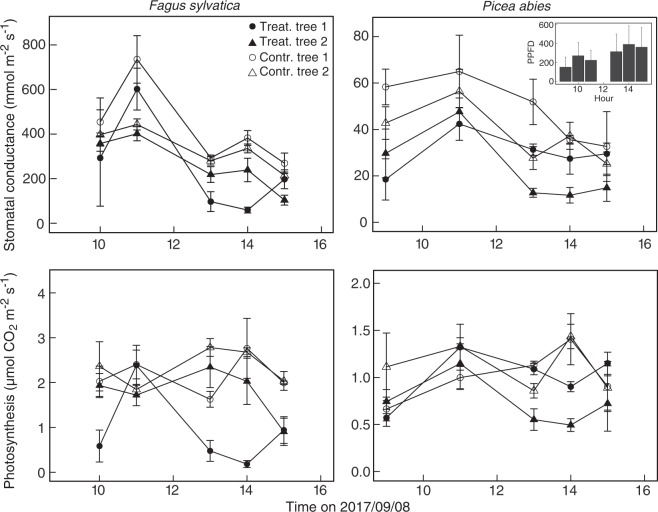
Stomatal conductance and net photosynthesis of the two treated and two control individuals of the two species 25 days after the cut was set (means ± SE). We continuously measured three sun-exposed shoots per tree and hour throughout the day (n = 3) with a LI-6400 from a gondola on a canopy crane. We found no statistical differences among treatment and control trees at each measurement time. Inlet graph shows Photosynthetic photon flux density (PPFD) over the measurement day.

On the day of the cut, data for leaf conductance to water vapour were available for Fagus only (leaf porometer data), which showed a slight reduction (n.s.) in leaf conductance in the two cut trees on this sunny day (Supporting Fig. 3). The same was found under bright weather in early September for both *Fagus* and *Picea* (Fig. 3). In *Fagus*, δ^13^C analyses of leaf cellulose indicated that one of the treatment trees had a consistently higher ^13^C discrimination, and, therefore, most likely lower g, even before the treatment (Supporting Fig. 4). 25 days after the cut, both *Fagus* and *Picea* were checked for leaf conductance and photosynthesis using a branch chamber that was suitable for both species (Fig. 3). These data confirmed the persistent but very small reduction in leaf conductance in response to the cut in *Fagus* and revealed no difference between treatment and control for *Picea*. The rates of leaf net-photosynthesis were not significantly affected in either species, as well.

**Figure 4:**
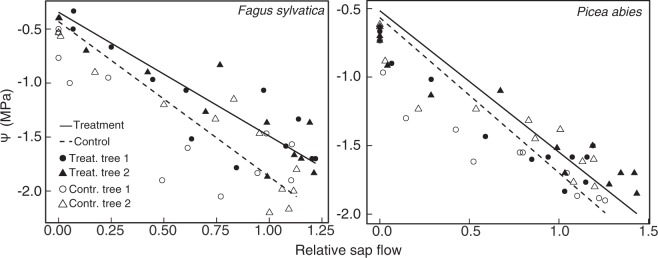
Mean shoot water potential (n = three branches per tree) in the canopy plotted against hourly mean relative sap flow (n = 1 sensor with 6 measurements per hour). The water potential at zero flux represents pre-dawn conditions plus the hydrostatic compound at 35 m height (−0.35 MPa). Sap flow was normalized on pre-treatment values. Linear regressions were significant at *p* < 0.001 and did not significantly differ among trees (two way-ANCOVA).

Plotting sap-flow against Ψ on the day of the cut revealed no distinct hysteresis in both treated and control trees (Supporting Fig. 5). We found no overall increase in hydraulic resistance in the treated trees (slope of a linear regression, cf. Fig. 4).

## Discussion

Interrupting half of the conduits of tall forest trees *in situ* by a chain saw cut did not reduce the water potential in the canopies of both species during warm summer weather. We observed a significant re-routing of sap flow in the stems (as predicted by Meidner and Sheriff in 1976^2^), with xylem near the cut edge being capable of a significantly higher conductivity (a 200% and 40% increase in sap flow in *Fagus* and *Picea* respectively). Hence, under ‘normal operation’ these conduits operate far below their capacity. Even under the high demand for sap flow under the given experimental conditions, losing half of the stem’s conductive area had no significant impact on canopy water relations.

The counter-intuitive lack of stress increment in the canopy of cut trees could reflect a relaxation of xylem tension immediately after cutting (the so-called ‘Iwanoff effect’^12,13^). However, the persistent lack of decreased water potential in response to the reduction in functional sapwood over the following weeks suggests that the trees had no problem meeting their water demand with half the conductive area even during warm summer weather.

Stomata in the cut trees slightly but not significantly closed in *Fagus* in response to the cut, but this had no significant effect on shoot net photosynthesis (A_n_). It is well known that small changes in g exert hardly any effect on photosynthesis when stomata are widely open, given that the maximum stomatal diffusion resistance represents only one fifth of the total resistance to CO_2_-uptake^14^. Thus, we conclude that the stomatal responses to a 50% loss of conductive stem cross-sectional area did not significantly affect tree carbon capture (cf. Supporting Fig. 6).

Previous theoretical considerations predicted a severe reduction of Ψ with decreasing conductive area, leading to a so-called ‘runaway embolism’^15^ or the ‘embolism cycle’^16^. The results of our treatment, however, show that downregulation of sap flux at 50% loss of sapwood area is very small and operates via stomata directly, with shoot water potential either unaffected or less rather than more negative. While *Fagus* was shown to reveal minimum canopy Ψ of down to −2.5 MPa at the same site during the summer drought of 2015^17^ (cf. Supporting Fig. 7) shoot water potential values measured at our site were far from this minimum value and even farther away from theoretically predicted values for catastrophic xylem failure (P_50_ *Fagus*: −3.8 MPa, P_50_ *Picea*: −4.0 MPa^18^). So, leaves must receive and process signals associated with the cut other than a permanent decrease in shoot Ψ. We can only assume that this signal is either of a chemical (e.g. release of abscisic acid [ABA] along the flow path) or physical (hydraulic)^19^ nature, or both. A brief reduction in leaf water status in a small fraction of the leaf tissue, like in the petiole or leaf veins, could trigger such a response (perhaps mediated by an ABA-release at leaf level^19^). Wounded tissue could exert a short-term signal but cannot explain the small and persistent effect as shown here^20^. Shaded leaves might have responded even less than the sun-exposed leaves we studied. Therefore, the overall crown transpiration might have undergone even less reduction as suggested by our stomatal conductance measurements from the canopy top. Finally, our sensors placed close to the cut edge indicate that the sensor directly opposite the cut did not capture the full speed of the re-routed sap flow, which occurred right near the edge, similar to what would happen at a barrier inserted into a river. We acknowledge, however, that in addition to the observed enhanced flow rates near the cut, water stored in the sapwood of the trees^21^ could have partly mitigated transport constraints during the day.

The results of this experiment suggest a considerable over-capacity in the conduit system of mature tree trunk xylem. Accordingly, the classical sap flow/Ψ plot revealed no overall increase in hydraulic resistance (slope of the regression, Fig. 4) which calls results of PLC measurements from micro CT-derived active conduit area into question^11^. Over-capacity in stem xylem is well known from practitioners in tree cultivation, who consider only a 90% or higher loss of bark and xylem as critical for tree survival^22^. Elazari-Volcani *et al*.^23^ found no effect on cell turgor by severe root pruning and stem incisions, and Scholander *et al*.^24^ assumed no significant impact on the water supply of a tropical liana when the stem was cut by half. This suggests that evolution of the conduit system selected for a high degree of insurance (redundancy), presumably not only accounting for mechanical damage, pathogen impact or damage by ground fires, but also for static demands.

In contrast to sporadic conduit failure by cavitation, cutting sapwood in half does reduce the potential conductive capacity across all conduit diameter classes, and thus, may represent a greater effective reduction of conductivity than under a drought-induced halving of conductivity (when flux is low and large vessels will quit first), because we also removed half of all smaller conduits that can be expected to remain intact under stress. Perhaps, branch xylem rather than trunk xylem represents a higher overall hydraulic constraint^16^, although this would be in conflict with the conservation of xylem cross-sectional area during branching (‘pipe model’^25^). A stem-cutting experiment in seedlings^26^ revealed no effects on g and Ψ of transpiring leaves when branches were cut by up to 90%. Only when two short-distance (2–8 cm) transverse cuts were made, the authors observed severe effects. Other studies employed even more drastic treatments (several overlapping cuts) causing leaf mortality^27,28^. Similarly, introducing artificial embolism in stem xylem of *Pinus ponderosa* seedlings, continuously decreased stomatal conductance but only affected Ψ when 99% of the xylem was embolized, with Ψ dropping by only 0.6 MPa from −1.5 to −2.1 MPa^19^. This drop in Ψ was far less than expected for a 50% or even 88% reduction in hydraulic conductance due to embolism^6,29^. Our study does not disprove the appropriateness of P_50_ values for measuring xylem hydraulic vulnerability, but we question the direct causal linkage between PLC_50_ (PLC_88_) and tree death, as it is often assumed. Our study was not performed under drought but we assume that a loss of 50% conduit area would have a much smaller effect under dry conditions when the water demand of the trees (not the atmosphere) would be far lower because of stomatal closure. Paradoxically, the demand for xylem conducting capacity declines as drought stress increases. We argue that cuticular transpiration alone (commonly <1% of peak sap flow in taxa from potentially dry areas, represented by minimum leaf diffusive conductance^7^) can be covered by only 1% of conduit capacity. Leaf shedding, a common phenomenon under severe to extreme drought in many species, would even reduce flux demand close to zero and exclude a loss of conductance driven by transpiration. Instead of representing a causality, xylem dehydration by 50% (embolization as a result of over-stretched capillary tension) could, therefore, also represent a side effect of general plant dehydration. Most metabolically active plant tissues, such as green foliage, die when the water content is reduced by 50%^30,31^.

It is difficult to explain a possible difference (if there is one) between a cut and a diffuse pattern of embolisms both arriving at 50% loss of conductive area. Any answer would need to account for hydraulic re-routing processes, which in case of a cut, were condensed to one bottle neck, with a high demand on transverse water flow over a small stem area. In case of cavitation, re-routing is spread over many micro-locations in the stem. An electric analogue would rank one big resistor similar to a sum of many small resistors (of the same total resistance), but when hydraulics come into play, a mix of serial and parallel resistors may hold a different answer. Cavitation is unlikely to block minor conduits (tracheids in angiosperms, late wood in gymnosperms) while our transverse cut interrupted all conduit types, including the narrow ‘safety routes’. Under drought, with most big conduits already cavitated, a cut as applied here would represent a severe constraint to the remaining hydraulic continuum, but any interruption of less than 90% is unlikely to affect supply in the leaf canopy, given the low demand.

In summary, our results for tall trees match the results of earlier branch cutting experiments that have used saplings, suggesting a lot of surplus conduit area in tree sapwood even under high flux conditions. Under drought-induced stomatal closure, when the demand for conductive capacity is minute, it is highly unlikely that a 50% failure of the hydraulic system is causally linked to tree mortality. The over-dimensioned xylem capacity may reflect mechanical needs, provide a safety margin in the case of pathogen impact (e.g. wilt diseases^32^) or fire damage. Under extreme drought stress, sap flow may become restricted to the small fraction of the narrowest non-embolized conduits, with no more than 1% of the stem cross-sectional area required. This seems to explain, why angiosperms retained tracheids, despite the higher transport capacity of vessels. The stem-system is, thus, unlikely to represent the critical transfer resistance under severe drought. Unlike commonly assumed^33^, the ultimate cause for a tree’s death during drought would rather be the lack of radial moisture flow towards the root surface in the soil matrix, and as a consequence, shoot tissue dehydration. Rooting depth and species- and tissue-specific dehydration tolerance may be more likely to explain vulnerability during drought, with large diameter conduit cavitation a by-product or symptom rather than a cause.

## Methods

### Study site and study species

The experiment was conducted at the Swiss Canopy Crane facility in a diverse mixed forest in the vicinity of Basel, Switzerland (47°28′N, 7°30′E), at an elevation of 550 m a.s.l. The forest is a mix of needle- and broad-leaved tree species with individuals about 130 years old and stocking on a rendzina-type soil on calcareous bedrock^34^. The climate is humid temperate with mean July temperatures of 19.2 °C and two thirds of the 900 mm mean annual precipitation falling during the growing season. Yet, the site is prone to summer drought^18,35,36^. This destructive experiment took place shortly before the study site was abandoned and the crane became dismantled. Weather data were available from the close weather service station in Binningen (c. 8 km distance; Supporting Fig. 8). On-site soil moisture was measured with a TDR probe (ML2 ThetaProbe, Delta-T Devices, Cambridge, UK) at −10 and −20 cm at three different spots within freshly trenched soil profiles of −20 cm depth. Throughout the whole study period, soil water content did not drop below 25% vol.

### Study design

We selected four similar sized trees of the species *Fagus sylvatica* and *Picea abies*, respectively, and determined two treatment and two control trees per species. With 8 trees in total, we reached the maximum of trees that could be monitored with sufficient temporal resolution from the crane’s gondola. On August 14th 2017, two trees of each species were cut with a chain saw 1.8 m above ground at the downwind side. After dye-testing for sapwood thickness (20–30 mm), we decided to place a cut 65 mm deep over half of the circumference. No further treatment was applied to the ca. 5 mm wide cut. Four weeks before the treatment, all trees were equipped with 20 mm long Granier-type sap-flow sensors (SFS2-M, UP GmbH, Ibbenbüren, Germany) 10 cm above the planned cut (white line, 1.8 m) and directly opposite the cut (Fig. 1). On the treatment trees, additional sap flow sensors at 3 m height were installed axially aligned above the lower two sensors. Later in the season, we added sap flow sensors 5 cm aside the cut and halfway between this sensor and the sensor opposite the cut in the treatment trees. The dead bark at the two insertion points for the sensor needles was carefully removed and aluminium sleeves were inserted into machine-drilled 20 mm deep holes at a 10-cm vertical distance from each other. Sensor needles were greased with a heat conducting paste, inserted into the sleeves and sealed with Teroson MS-930^©^ sealing adhesive from the outside. We protected the sensors from environmental influences with radiation shields made of bubble warp aluminium foil. Data were recorded from July 24^th^ until September 25^th^ every ten minutes with a sensor node (Channel Node, Decentlab GmbH, Dübendorf, Switzerland), wirelessly transmitted to a data logger (Base Station, Decentlab GmbH) and then broadcasted to a server via cellular network.

### Evaluation of sap flow

Sap flow data were analysed following Granier^37,38^. In order to estimate zero flux during night and account for night time transpiration, night time maxima in temperature difference (ΔT_max_) were adjusted using the maximum during a seven-day period. We also performed a correction of the temperature difference in the sapwood (ΔT_SW_) to account for the narrow sapwood in the investigated individuals^39^. For our analyses, we calculated relative sap flow values which were normalized by the mean of daily sap flow maxima during the pre-treatment period.

### Shoot water potential

We measured shoot water potential (Ψ) with a Scholander pressure chamber (Model 1000, PMS Instruments, Albany, OR, USA) on three randomly chosen terminal branches from the upper part of the sunlit crowns using the canopy crane. We cut ca. 15 cm long branchlets with two to four leaves (*Fagus*) or current-year shoots (*Picea*) with a razor blade immediately before measuring.

### Stomatal conductance and photosynthesis

In *Fagus*, stomatal conductance (g) was measured on five sun-exposed branches per tree with a Decagon SC-1 Leaf Porometer (Decagon Devices, Pullman, WA, USA) on the day of the treatment. However, the porometer could not be used with the small needles of *Picea*. Therefore, we measured stomatal conductance and photosynthesis in both *Fagus* and *Picea* with the conifer chamber of the LI-6400XT gas exchange system (LI-COR, Lincoln, NE, USA) 25 days after the treatment, providing stomatal conductance and photosynthesis values for both *Fagus* and *Picea*. Because this system is much slower than the leaf porometer, we studied only three sun-exposed branches per tree at each measuring time. Photosynthetically active radiation ranged from 100 to 750 µmol m^−2^ s^−1^ and averaged at 300 µmol m^−2^ s^−1^ across the day. Leaves (*Fagus*) and shoots (their needles, *Picea*) were analysed for leaf area in the lab. We calculated single (projected) leaf area from weight using a species- and site-specific conversion factor for the weight/leaf area relationship in both species.

### Sapwood depth

Sapwood depth was determined with an ink trial. One 10 cm deep core was taken with an increment corer on each tree and the hole was immediately filled with a solution of black ink and sealed with Teroson MS-930^©^ sealing adhesive. To guarantee a sufficient filling of the hole after sealing, more ink was injected through the sealing with a syringe and a needle until the ink started sprinkling out of the puncture so that the hole was completely filled with ink. The puncture was sealed again straightaway. After half an hour, we took increment cores 1 cm above the filled holes and measured the length of the core that was infiltrated with the black ink solution. From earlier work, we know that conducting sapwood depth in comparably-sized trees of *Fagus sylvatica* and *Picea abies* is about 6 and 4.5 cm, respectively^40,41^.

### Cellulose extraction

Cellulose extraction was done on five current-year leaves from different parts of the canopy of each individual of the two species after the protocol by Brendel^42^. Leaves were harvested on 9 September 2017, taken to the lab, dried for 24 hours at 75 °C, and ground finely. The resulting powder was processed in filter bags (ANKOM Technology, Macedon, NY, USA), lipids and sugars were extracted by washing with toluene:ethanol (2:1) and DI water. Samples were bleached in an ultrasonic cleaner with an aqueous sodium chlorite/glacial acetic acid solution, rinsed with DI water afterwards, and dried at 50 °C for two days.

### δ^13^C measurements

Dry powder obtained from extracted cellulose of 5 sunlit current-year leaves per individual from the upper part of the canopy was transferred into tin capsules and was analyzed in a Flash 2000 elemental analyzer coupled to a Delta V Plus continuous-flow isotope ratio mass spectrometer (IRMS) via a Conflo IV interface (Thermo Fisher Scientific, Bremen, Germany). Samples went through flash combustion at ca. 1800 °C in the presence of oxygen, before the emerging CO_2_ was fed into the IRMS. Stable isotope data were expressed in the delta notation (δ^13^C), relative to the ^12^C/^13^C ratio of Vienna Pee Dee Belemnite standard (R_VPDB = 0.0111797).

### Non-structural carbohydrates

NSC (i.e., starch, sucrose, fructose and glucose) were quantified in current-year leaves of sun-exposed branches in each individual of the two species (n = 4 leaves per individual). Samples were collected on 9 September 2017, two weeks after the treatment took place. For chemical analysis in the lab, we used a modified protocol for enzymatic-photometric determination of low molecular weight sugars and starch^43,44^.

### Statistics

We used R statistical software (http://cran.r-project.org) to test the difference between control and treatment trees applying a two-sample t-test as well as a Tukey’s range test in combination with an ANOVA (post-hoc) for each pair of data points. For diurnal sap flow analysis, we divided the sap flow measurements into three time periods (5–10 h, 10–15 h and 15–20 h) and checked for significant differences between treatment and control with a two-sample t-test. Linear regression was used to investigate the relationship between hourly sap flow and hourly Ψ and slopes of the regressions were compared by ANCOVA.

## Electronic supplementary material

Supporting Information

## Data Availability

The datasets generated during and/or analyzed during the current study are available from the corresponding author on reasonable request.
