# Supplementary material for: Losing half the conductive area hardly impacts the water status of mature trees
Source: Sci Rep. 2018 Oct 9;8:15006. doi: 10.1038/s41598-018-33465-0 (PMC6177434; doi:10.1038/s41598-018-33465-0)
Supplement: Supplementary file 1 — Supporting Information [file 41598_2018_33465_MOESM1_ESM.docx]

## *Scientific Reports*

## Supporting Information

Losing half the conductive area hardly impacts the water status of mature trees

Authors: Lars Dietrich, Günter Hoch, Ansgar Kahmen, Christian Körner

The following Supporting Information is available for this article:

Supporting Figure 1 Mean conducting sapwood depth in the four individuals of the respective study species (n = 4 ± SD). Sapwood depth was determined with an ink injection trial where a 10-cm deep wood core was taken with an increment corer and the remaining hole immediately filled with black ink. After half an hour, a second core was taken ca. 1 cm axially above the first hole and the dyed part of the core was measured.

Supporting Figure 2 Pre- and post-treatment sap flow for the different sensors on the investigated trees (n=2 trees). The upper two panels compare sensors between treatment and control, the lower panel shows the two sensors at 3 m height on the treated individuals. Sap flow was standardized on the mean of the maxima of the respective sensor on the respective tree during the two-week pre-treatment period. The horizontal clack line marks the beginning of the treatment (cut).

Supporting Figure 3 Stomatal conductance of the two treated and the two non-treated individuals of *Fagus sylvatica* during the first day of the experiment (n = 3 leaves per tree ± SE). We continuously measured five sun-exposed leaves per tree and hour from a gondola on a canopy crane throughout the day.

Supporting Figure 4 δ^13^C of the treatment and control trees’ leaf cellulose (n = 5, means ± SD). Cellulose δ^13^C is independent from the treatment of the trees in this experiment since leaves were built long before the experiment took place. We only found a statistical different value of δ^13^C in Fagus2. Significances were obtained from a posthoc Tukey’s honest significance test.

Supporting Figure 5 Hourly mean shoot water potential (n = 3 branches per tree) plotted versus hourly mean relative sap flow (n = 6 measurements of 1 sensor per hour) of treated and control trees on the day of the cut. The cut was set at shortly before 9 a.m. Daytime is indicated by numbers on a two-hour interval. Not distinct hysteresis could be detected, neither in treated nor in control trees.

Supporting Figure 6 Mean non-structural carbohydrate (NSC) contents of the two treated and untreated trees per species (n = 2). NSC were measured in four current-year leaves from different sunlit branches in the upper crown of each tree which were harvested on 9 September 2017 (14 days after cutting, DOY 240) and dried for 24 h at 75°C.

Supporting Figure 7 Boxplots of midday (n = 17) and pre-dawn (n = 9) shoot water potential of the study trees during the dry summer of 2015. Treatment trees are indicated by grey boxes. We only found a statistical difference between pre-dawn water potentials of Fagus1 and Fagus4 in a posthoc Tukey’s honest significance test.


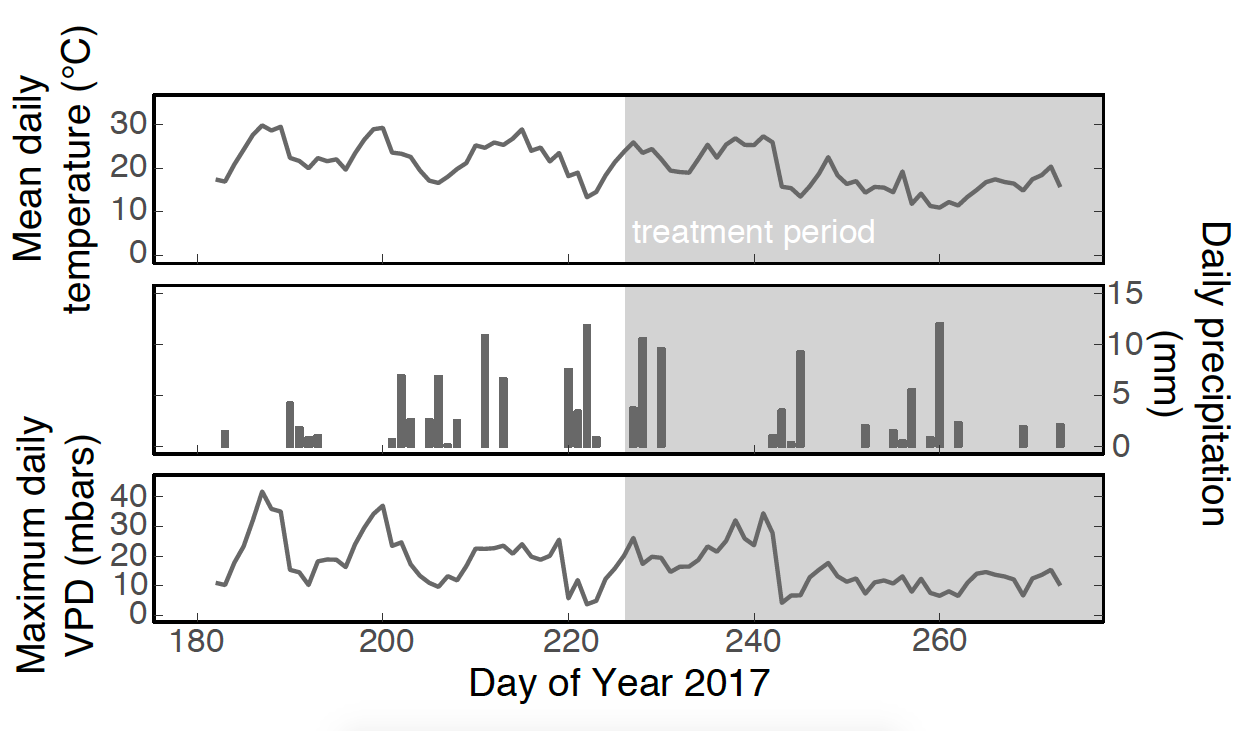


Supporting Figure 8 Environmental conditions at the MeteoSwiss weather station in Basel Binningen in direct vicinity of the study site during the experimental period and the preceding weeks. The period after the cutting took place is highlighted by a grey box. Rainfalls at the beginning of the treatment period happened during the night.
